# Role of Sex Hormone Levels and Psychological Stress in the Pathogenesis of Autoimmune Diseases

**DOI:** 10.7759/cureus.1315

**Published:** 2017-06-05

**Authors:** Salman Assad, Hamza H Khan, Haider Ghazanfar, Zarak H Khan, Salman Mansoor, Muhammad A Rahman, Ghulam H Khan, Bilal Zafar, Usman Tariq, Shuja A Malik

**Affiliations:** 1 Department of Medicine, Shifa International Hospital, Islamabad, Pakistan; 2 Graduate, Shifa International Hospital, Islamabad, Pakistan; 3 Department of Internal Medicine, Shifa International Hospital, Islamabad, Pakistan; 4 Department of Medicine, Shifa College of Medicine; 5 Department of Neurology, Shifa International Hospital, Islamabad, Pakistan; 6 Student, Shifa International Hospital, Islamabad, Pakistan; 7 Internal Medicine, Shifa College of Medicine

**Keywords:** central tolerance, autoimmunity, systemic lupus erythematosus, multiple sclerosis, peripheral tolerance

## Abstract

The aim of this review article is to assess the connection between psychological stress and sex hormones and their effect on the development of autoimmune diseases. Psychological stress describes what people feel when they are under mental, physical, or emotional pressure. We searched for online articles using MEDLINE®, Embase, Cochrane Library and Google Scholar. Our research yielded a total of 165 articles out of which 30 articles were considered for further perusal. The articles were reviewed from February 2016 to February 2017. Case reports and patients suffering from hematolymphoid malignancies and active infections were excluded from the review. Estrogen and testosterone are potential physiological regulatory factors for the peripheral development of CD4+CD25+ T regulatory cells. Stress at any age leads to the depletion of estrogen and testosterone stores in the body, leading to the loss of expansion of T regulatory cells, making the immature B cells evade the negative selection at the germinal center, or in other words, leading to the loss of central tolerance, a triggering event in autoimmune diseases like systemic lupus erythematosus. Autoimmune diseases in women are most likely due to changes in estrogen levels during mental, physical, pre-menopausal, post-menopausal, and pregnancy-induced stress. We conclude that modulating estrogen in females (pre-menopausal and post-menopausal) and testosterone in males can be used to treat stress-related immune imbalance resulting in autoimmune diseases in both sexes.

## Introduction and background

Autoimmunity is one of the top 10 causes of death in women under 65 years of age. According to the Autoimmune Related Diseases Association (AARDA), it is estimated that 50 million Americans have an autoimmune disease based on the National Institute of Health (NIH) epidemiology studies as well as individual patient group data from members of the National Coalition of Autoimmune Patient Groups (NCAPG). There is an increase in the prevalence of autoimmune diseases in the older age group [1-2]. It has been observed that women are affected more than men. One of the prevalent causes of autoimmunity is a loss of central or peripheral tolerance. This article mainly emphasizes the hormonal and stressful conditions which make women more sensitive to autoimmune diseases. Patients with immune-based diseases, such as multiple sclerosis (MS), asthma, or systemic lupus erythematosus (SLE), may have exacerbations during the menstrual period (of the menstrual cycle) or during pregnancy. Hormonal levels are affected during pregnancy, especially estrogen, which remains low during pregnancy but peaks near term. Similarly, there is a higher association of autoimmune disorders after menopause linked to lower blood estrogen levels [3].

A study concluded that the marginal zone B cells are capable of secreting a high-affinity anti-DNA antibody, which leads to immune complex deposition [2]. An elevation in estrogen levels alters the strength of B-cell receptor (BCR) signaling, resulting in the diminished negative selection of potentially pathogenic B cells. On the other hand, naturally occurring CD4+CD25+ regulatory T cells play an important role in mediating maternal tolerance to the fetus during pregnancy, and this effect might be regulated via maternal estrogen secretion. Although estrogen concentration in the pharmaceutical range has been shown to drive the expansion of CD4+CD25+ regulatory cells, estrogen at physiological doses, in addition to expanding regulatory T cells in different tissues, also enhances expression of the Foxp3 gene (a hallmark for CD4+CD25+ regulatory T cell function) and the IL-10 gene. Such converted CD4+CD25+ T cells have a similar regulatory function as naturally occurring regulatory T cells, as demonstrated by their ability to suppress naive T cell proliferation in a mixed lymphocyte reaction (MLR). The estrogen receptor is present in the CD4+CD25- T cells, and the conversion of CD4+CD25- T cells into CD4+CD25+ T cells stimulated by estrogen could be inhibited by a specific inhibitor of estrogen. More irregularities are expected with the BCR modulation in pre-menopausal women because of a concomitant increase in expression of CD4+CD25+ T regulatory cells and estrogen levels. Post-menopausal women have decreased expression of CD 4+ CD25+ T regulatory cells due to low estrogen levels. This can trigger an autoimmune response with the evasion of highly reactive T cells to self-antigens. Studies done on males concluded that increased testosterone levels have been associated with an increased expression of expression of CD 4+ CD25+ T regulatory cells [3]. Psychological stress may aggravate the natural fall in estrogen during the menstrual cycle and reduce peak levels. Similarly, it remains unclear whether the drop in testosterone levels during exposure to mental stress is caused by decreased luteinizing hormone (LH) secretion or an inadequate response at the pituitary level. The decreased estrogen levels in young women and post-menopausal women, and reduced testosterone levels in men, results in the decreased expansion of regulatory T cells making them prone to autoimmune diseases. The antibodies believed to mediate pathogenesis in lupus display somatic mutations, and have undergone isotype class switching. Stress reducing measures should be adopted in females as well as in men to decrease the expression of autoimmunity events and increase the levels of regulatory T cells. Testosterone levels remain higher throughout a male’s lifetime and do not follow alterations as in females; therefore, males are less prone to autoimmune events than females.

## Review

The aim of this review article was to assess the psychological stress-related connection with sex hormones and their effect on the development of autoimmune diseases. We searched for online articles using MEDLINE®, Embase, Cochrane Library and Google Scholar. The keywords/phrases used were: “autoimmune diseases epidemiology”, “humans”, “incidence”, “sex characteristics”, “autoimmune diseases immunology”, “psychological stress”, “central tolerance”, “peripheral tolerance”, “T-lymphocytes/immunology”, “estradiol”, “testosterone”, “lymphocyte activation”, “pregnancy immunology” and “cytokines”. Articles published between 1978-2017 related to these topics of interest, and included both genders (age range: 20-99 years), pre-menopausal and post-menopausal women, the population suffering from autoimmune diseases, the population who underwent any psychosocial stressor in the previous three months, and the population involved in mild to moderate physical exercise during the previous three months. The articles were reviewed from February 2016 to February 2017. A total of 165 articles fulfilling the inclusion criteria were reviewed and analyzed, out of which 30 articles were included in this study. Case reports and patients suffering from hematolymphoid malignancies and active infections were excluded from the review.

### Self-renewal of embryonic stem cells

A study conducted to observe the self-renewal of embryonic stem cells (ESCs) concluded that ESCs can self-renew and differentiate into all cell lineages. Calcium is a universal second messenger which regulates a number of cellular pathways. Store-operated calcium channels (SOCCs), but not voltage-operated calcium channels, are present in mouse ESCs (mESCs). In this study, store-operated calcium entry (SOCE) was found to exist in mESCs. It was observed that 17β-estradiol (E2) enhanced mESC proliferation. Pluripotent markers, Sox-2, Klf-4, and Nanog were down-regulated by 2-aminoethoxydiphenyl borate (2-APB). 2-APB is a reliable blocker of store-operated Ca2+ entry but an inconsistent inhibitor of InsP3-induced Ca2+ release, suggesting that the self-renewal property of mESCs relies on SOCE. SOCC blockers can reverse both stimulated proliferation and increased SOCE, suggesting that E2 mediates its stimulatory effect on proliferation via enhancing SOCE [1].

### Estrogen-mediated maternal tolerance during pregnancy

Naturally occurring CD4+CD25+ regulatory T cells play an important role in mediating maternal tolerance to the fetus during pregnancy, and this effect might be regulated via maternal estrogen secretion. Although estrogen concentration in the pharmaceutical range has been shown to drive the expansion of CD4+CD25+ regulatory T cells; estrogen at physiological doses, in addition to expanding T regulatory cells in different tissues, also enhances expression of the Foxp3 gene (a hallmark for CD4+CD25+ T regulatory cell function) and the IL-10 gene. Such converted CD4+CD25+ T cells have a similar regulatory function as naturally occurring regulatory T cells, as demonstrated by their ability to suppress naive T cell proliferation in a mixed lymphocyte reaction. The estrogen receptor is present in the CD4+CD25- T cells and the conversion of CD4+CD25- T cells into CD4+CD25+ T cells stimulated by estrogen could be inhibited by ICI 182,780, a specific inhibitor of ERs (endoplasmic reticulum) [2]. It is generally accepted that the conventional mature follicular and marginal zone B cell subsets are derived from the transitional B cell pool. There is some evidence for a branch position at the late transitional stage toward pre-follicular and pre-marginal zone B cells; however, the presence of such precursors requires further investigation [2]. Studies in which the expression of specific molecules of the BCR signaling cascade is altered have clearly shown that the strength of the BCR signal helps determine the fate of B cell maturation [3]. Manipulation of the BCR to produce very potent signals favors the development of the non-conventional B cell population (which arises through mechanisms distinct from the conventional B cell subsets) [4]. On the other hand, a modest decrease in signaling strength favors the development of follicular B cells. Finally, a strongly diminished BCR signal favors the development of marginal zone B cells [5]. Thus, a general paradigm has emerged in which a strong BCR signal favors B1 B cells (the cells with no memory and that undergo self-renewal in the periphery), a strong-to-intermediate signal favors follicular B cells, and a weak signal favors marginal zone B cells. However, there are some exceptions to this model. For example, mice deficient in the phosphatase and tensin homolog PTEN, which down-regulates CD19-mediated activation of phosphoinositide 3-kinase, exhibit an expansion of both marginal zone and B1 B cells [6]. There is experimental evidence that the surface density of the BCR itself can alter the development of B cell subsets [7]. Also, the specificity of the BCR may skew the development of mature subsets. According to one study, B cells of transgenic mice that expressed an anti-phosphorylcholine Ab showed preferentially development into marginal zone and B1 B cells (B cells with no memory) [8]. Although, it can be argued that the degree of BCR cross-linking, and not Ag specificity, is responsible for altering of the naive repertoire to distinct B cell subsets [9].

### Negative selection

An auto-reactive BCR in the presence of self-Ag (antigens) is barred from entry into the mature, immune-competent B cell repertoire. This is accomplished by three distinct mechanisms of tolerance induction: receptor editing, deletion, or anergy induction. BCR engagement of immature B cells can result in the reactivation of the recombinase machinery that triggers a new specificity for B cells that is not auto-reactive [10]. B cells that successfully undergo receptor editing may be spared from elimination [11]. Immature B cells will be deleted via apoptosis if the auto-reactive specificity is not extinguished [12]. Auto-reactive B cells with less extensive BCR cross-linking are rendered anergic [13]. These cells do not undergo rapid cell death but are non-responsive to BCR engagement. The exact mechanisms by which the BCR determines the mode of negative selection have yet to be clearly established. It still remains to be determined how BCR ligation can induce negative selection of immature B cells and also activation of mature B cells. No major difference in the molecules of the BCR signal transduction pathway has been observed in the comparison of anti-IgM-treated immature and mature B cells in vitro. However, subtle differences such as increased calcium immobilization, an increase in the breakdown of inositol 1,4,5-trisphosphate, and enhanced phosphorylation of regulatory tyrosine residues have been reported and may be responsible for determining whether an apoptotic or activation program is initiated [14]. In addition, the BCR of mature B cells enters into lipid rafts following Ag engagement, whereas the BCR of immature B cells is excluded [15]. Other differences between immature and mature B cells that may affect the outcome of BCR signaling include decreased levels of the negative regulator CD22 and increased levels of the positive regulators, B cell linker protein, Btk, and phospholipase Cγ, in immature B cells [14]. There are several reports demonstrating the sensitivity of transitional type 1 B (T1 B) cells in vitro to BCR-induced apoptosis, whereas T2 B cells (transitional type 2 B cells) are resistant to it [16]. Other studies suggest that both T1 and T2 B cells are sensitive to BCR-mediated apoptosis, but only T2 B cells can be rescued by T cell help (CD40 and IL-4) [17]. The ability of T3 B cells (transitional type 3 B cells) to undergo BCR-mediated apoptosis remains to be determined. There is now increasing evidence that transitional B cells of the spleen are also the target of negative selection [18]. Not all auto-reactive B cells are eliminated from the pre-immune inventory. Cells with minimal interaction with self-antigens are ignored either because of the low-affinity of the BCR or the low concentration of accessible antigen at the immature and transitional B cell stages. These remain viable in the periphery. Once these low-affinity B cells become activated, they undergo a process of somatic mutation and affinity maturation to become high-affinity B cells. During a germinal center response, these newly arising high-affinity auto-reactive B cells are subject to elimination. This has been demonstrated as their rescue by increased expression of endogenous or exogenous Bcl-2 [19]. It is not yet clear how their elimination occurs, although it can be postulated that it requires Ag engagement of the BCR in the absence of T cell help [20]. The antibodies are believed to mediate pathogenesis in lupus display somatic mutations and have undergone isotype class switching [21]. In an autoimmune-prone population, the BCR-mediated selection events that block the emergence of auto-reactive B cell at the germinal center stage are impaired. It is not known whether there can be a defect in germinal center selection without a defect in the selection of immature cells as the mechanisms of germinal center selection are poorly understood.

### Modulation of strength of BCR by estrogen and testosterone

Systemic lupus erythematosus (SLE) affects females 10 times more frequently than males and strikes predominantly during the reproductive years. It also displays an earlier onset of disease and a shortened lifespan in females as compared with males [22]. Administration of E2, a highly active metabolite of estrogen, accelerates disease onset in females, whereas administration of testosterone ameliorates disease progression [22]. These studies provided compelling evidence that an alteration in sex hormones levels is one of the factors that might contribute to the predominance of SLE in the female population. Normally, immune tolerance is maintained through the deletion of the DNA-reactive B cells that arise in the immature repertoire. Estrogen administration is sufficient to break tolerance of high-affinity DNA-reactive B cells. The estrogen-treated population displayed a lupus phenotype characterized by a rise in serum anti-DNA titers, glomerular immune complex deposition, and expansion and activation of DNA-reactive B cells [23]. Examination of the B cell subsets in estrogen-treated groups revealed several interesting findings. First, a reduction in the number of transitional B cells was observed. This was in agreement with earlier studies that demonstrated that B cell lymphopoiesis is reduced in both pregnant and in the estrogen-treated population [24]. An alteration in the ratio of transitional T1: A T2 B cell was also observed due to the relative increase in T2 cells in estrogen-treated groups [24]. In addition, BCR-mediated apoptosis is reduced in the transitional B cell population. These data confirmed impaired negative selection occurring at the transitional B cell stage. Analysis of B cells separated from the E2-treated population shows that DNA-reactive marginal zone B cells, but not the follicular B cells, are spontaneously activated [24]. Thus, these results affirm that the marginal zone B cells are capable of secreting high-affinity anti-DNA antibodies, which can lead to immune complex deposition. A single-cell polymerase chain reaction is done to analyze the repertoires of transitional and mature B cells. It showed that high-affinity anti-DNA B cells are routinely eliminated at both the immature and transitional B cell stages in normal mice. However, in E2-treated-mice (estrogen-treated mice), high-affinity DNA-reactive B cells are rescued from negative selection and mature to immune-competence. Additionally, the high-affinity anti-DNA B cells out-compete the low-affinity anti-DNA B cells for entry into the mature B cell pool. Such results led to the proposition that an elevation in estrogen levels alters the strength of BCR signaling such that there is a diminished negative selection of potentially pathogenic B cells.

Estrogen receptors, ERα and ERβ, are expressed in B cells, demonstrating that the B cell is, in fact, a target for the action of estrogen. These receptors act as transcription factors to regulate the expression of numerous target genes. To analyze the molecular effects of estrogen on B cells, microarray analysis and subtractive hybridization were done and it showed that the BCL-2 gene is directly E2 responsive and its increased expression in B cells has been shown to disturb the negative selection of auto-reactive B cells; however, increased BCL-2 expression alone is not sufficient to induce a fulminant autoimmune phenotype [25]. The increase of CD22 and SHP-1 in E2-treated mice was an intriguing finding because the conclusions are drawn from studies of CD22- and SHP-1-deficient population, which suggests that the reduced expression of these molecules leads to the activation of auto-reactive B cells. It might be possible that a weakened BCR signal, resulting from increased CD22 and/or SHP-1 levels, would favor the escape of B cells from the negative selection. The weaker BCR signal created by over-expression of CD22 or SHP-1 may be responsible for the diminished susceptibility to BCR-mediated apoptosis of transitional cells and for the expansion of marginal zone B cells. Testosterone supplementation exerts a versatile protective effect during experimental autoimmune orchitis (EAO) development. In the testes itself, testosterone appears to induce an expansion of suppressive regulatory T cells from naive T cells leading to an enhanced representation of regulatory T cells within the CD4+ T cell population, while simultaneously inhibiting the synthesis of pro-inflammatory mediators TNF-α, and IL-10. Clearly, the exact proportion of systemic and local actions of testosterone requires further investigation. Taken together, these findings suggest that testosterone plays an important role in the maintenance of immunological balance in the testes, and point to a previously unrecognized role of testosterone in the differentiation of regulatory T cells [26].

### Stress and hormonal levels

By definition, stress is any uncomfortable "emotional experience accompanied by predictable biochemical, physiological and behavioral changes” [27]. Psychological stress describes what people feel when they are under mental, physical, or emotional pressure. Psychological stress may aggravate natural falls in estrogen during the menstrual cycle and reduce peak levels. The decreased estrogen levels in younger women and post-menopausal women result in decreased expansion of regulatory cells. This makes them prone to autoimmune diseases [28]. Stressful situations as experienced during work, before tournaments, or while anticipating exams, have been shown to decrease testosterone levels. Stress release, on the contrary, can have an elevating effect on androgen levels. The same effect was seen in men undergoing workplace re-organization and threatened by unemployment. After the workplace situation improved, testosterone levels clearly increased; however, there was a marked variation between the subjects [29]. It remains unclear whether the drop in testosterone levels (in exposure to mental stress) is caused by decreased luteinizing hormone secretion or a lack of adequate response at the pituitary level. In settings combining the mental and physical aspects of stress, testosterone can drop to hypogonadal levels. The decrease of testosterone levels under stressful situations is usually not sufficiently compensated by the pituitary [30].The etiology of autoimmune diseases is multifactorial: genetic, environmental, hormonal, and immunological factors are all considered important in their development. The majority of the autoimmune disorders are due to idiopathic factors. Many retrospective studies concluded that a high proportion (up to 80%) of patients reported uncommon emotional stress before disease onset. Recent reviews discuss the possible role of psychological stress, and of the major stress-related hormones, in the pathogenesis of autoimmune disease. It is presumed that the stress-triggered neuroendocrine hormones lead to immune dysregulation, which ultimately results in autoimmune disease, by altering or amplifying cytokine production. The treatment of autoimmune disease should thus include stress management and behavioral intervention to prevent stress-related immune imbalance.

## Conclusions

We conclude that modulating estrogen in females (pre-menopausal and post-menopausal) and testosterone in males can be used to treat stress-related immune imbalance resulting in autoimmune diseases in both sexes. Testosterone and estrogen have obvious effects on the expansion of regulatory T cells. Stress reducing measures should be adopted in females as well as in males to decrease the expression of autoimmunity events. Stress reduces testosterone and estrogen levels leading to a decreased expansion of regulatory T cells, which are effective for controlling the expression of autoreactive T cells and B cells. It is most likely that the disruption of this mechanism occurs at the levels of central tolerance.
